# Supplementary material for: A Quantitative Evaluation of COVID-19 Epidemiological Models
Source: medRxiv. 2021 Feb 8:2021.02.06.21251276. Preprint. [Version 1] doi: 10.1101/2021.02.06.21251276 (PMC7872378; doi:10.1101/2021.02.06.21251276)
Supplement: 1 [file NIHPP2021.02.06.21251276-supplement-1.pdf]

medRxiv preprint doi: https://doi.org/10.1101/2021.02.06.21251276; this version posted February 8, 2021. The copyright holder for this preprint (which was not certified by peer review) is the author/funder, who has granted medRxiv a license to display the preprint in perpetuity.
This article is a US Government work. It is not subject to copyright under 17 USC 105 and is also made available for use under a CC0 license.

Supplementary 1: Supplementary Figures for "A Quantitative Evaluation of COVID-19 Epidemiological Models"

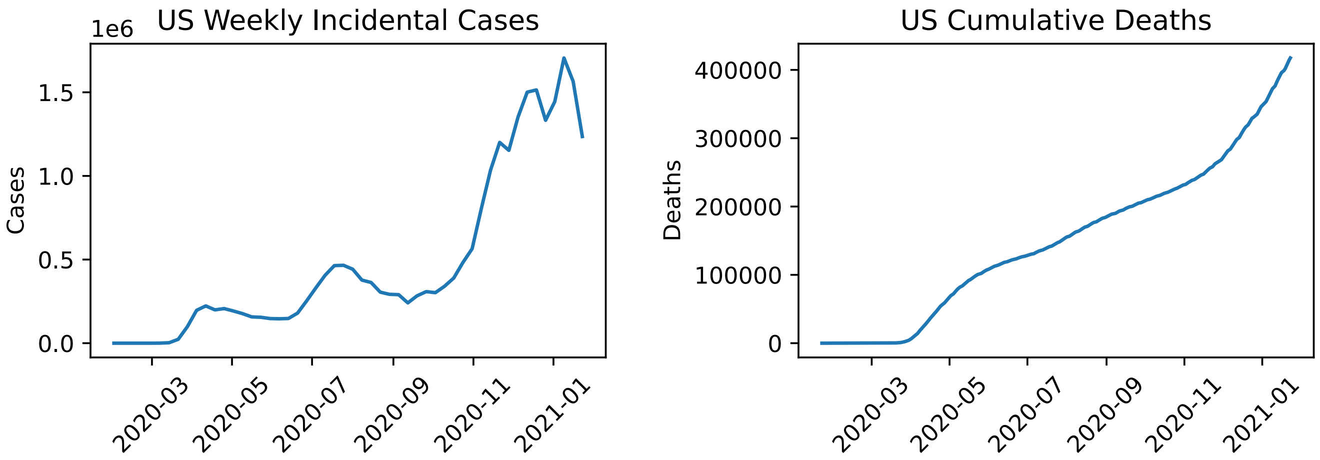

Fig. S1. COVID-19 in the US.

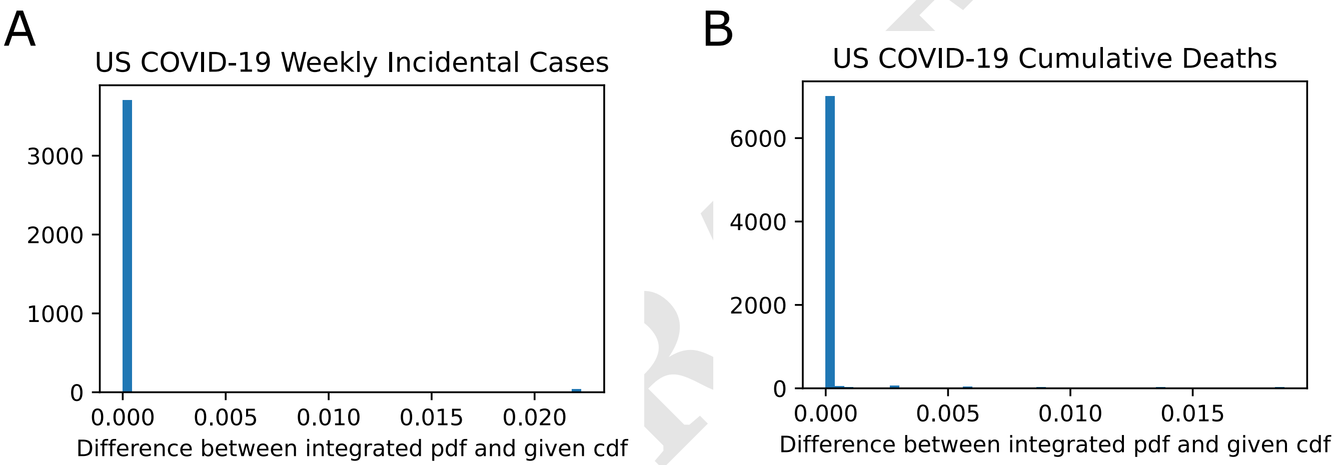

Fig. S2. Overall quality of CDF to PDF Conversion.

medRxiv preprint doi: https://doi.org/10.1101/2021.02.06.21251276; this version posted February 8, 2021. The copyright holder for this preprint (which was not certified by peer review) is the author/funder, who has granted medRxiv a license to display the preprint in perpetuity.
This article is a US Government work. It is not subject to copyright under 17 USC 105 and is also made available for use under a CC0 license.

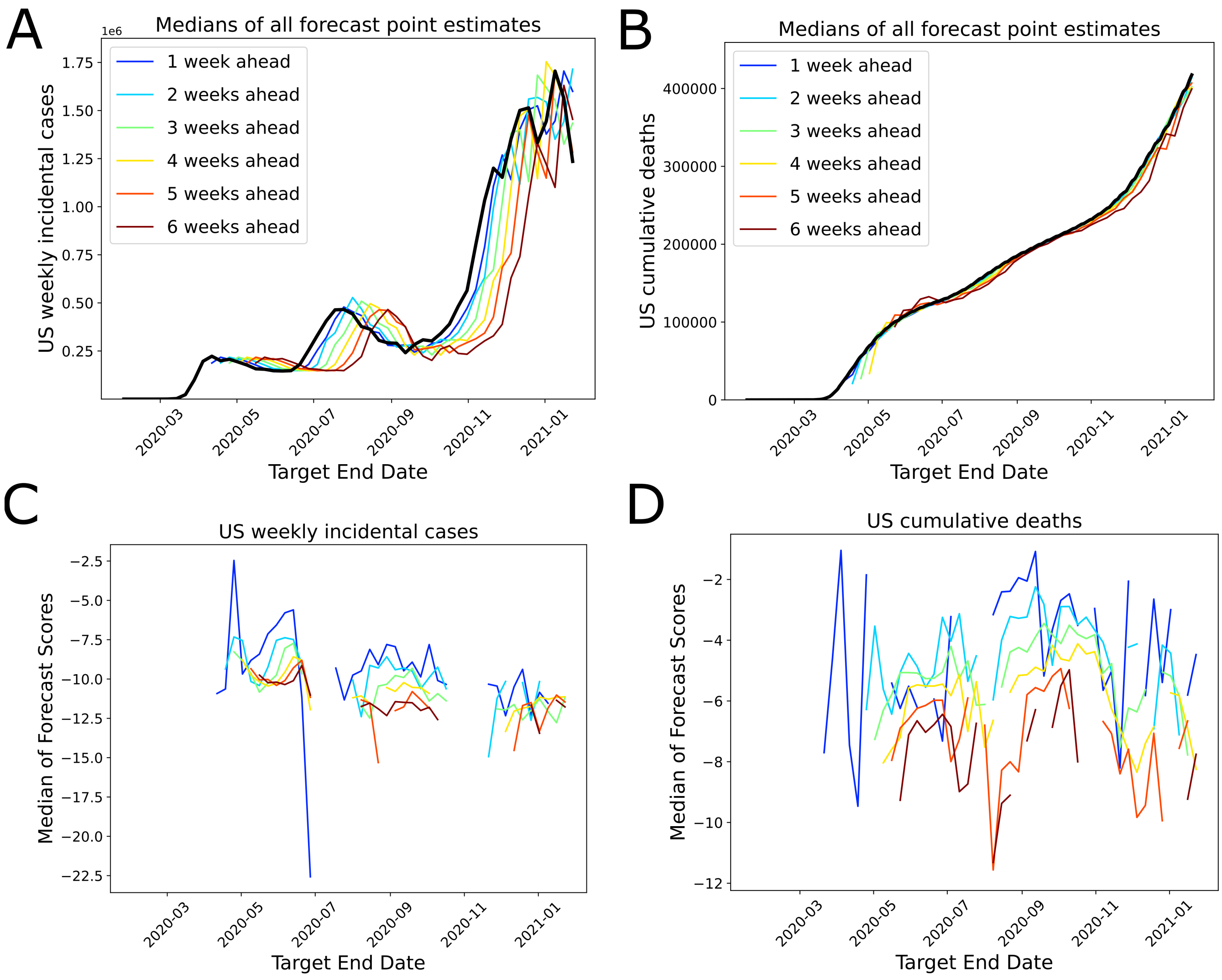

Fig. S3. Model forecast performances over time. A. Black solid curve represents the observed US weekly incidental case counts. Other curves represent the median of the forecasts for the target end date made from 1 to 6-weeks prior to the target end dates. B. Black solid curve represents the observed US cumulative death counts. Other curves represent the median of the forecasts for the target end date made from 1 to 6-weeks prior to the target end dates. C. Curves represent the median of the forecast scores colored based on their forecasting horizon (1-week prior to 6-weeks prior color-matching to sub-panel A). D. Curves represent the median of the forecast scores colored based on their forecasting horizon (1 to 6-weeks prior, with colors matching to sub-panel B). Discontinuities in the score plots imply that the median value of the scores for a particular time-horizon is $-\infty$, demonstrating the poor performance especially on the inflection points of the epidemiological curves.

medRxiv preprint doi: https://doi.org/10.1101/2021.02.06.21251276; this version posted February 8, 2021. The copyright holder for this preprint (which was not certified by peer review) is the author/funder, who has granted medRxiv a license to display the preprint in perpetuity. This article is a US Government work. It is not subject to copyright under 17 USC 105 and is also made available for use under a CC0 license.

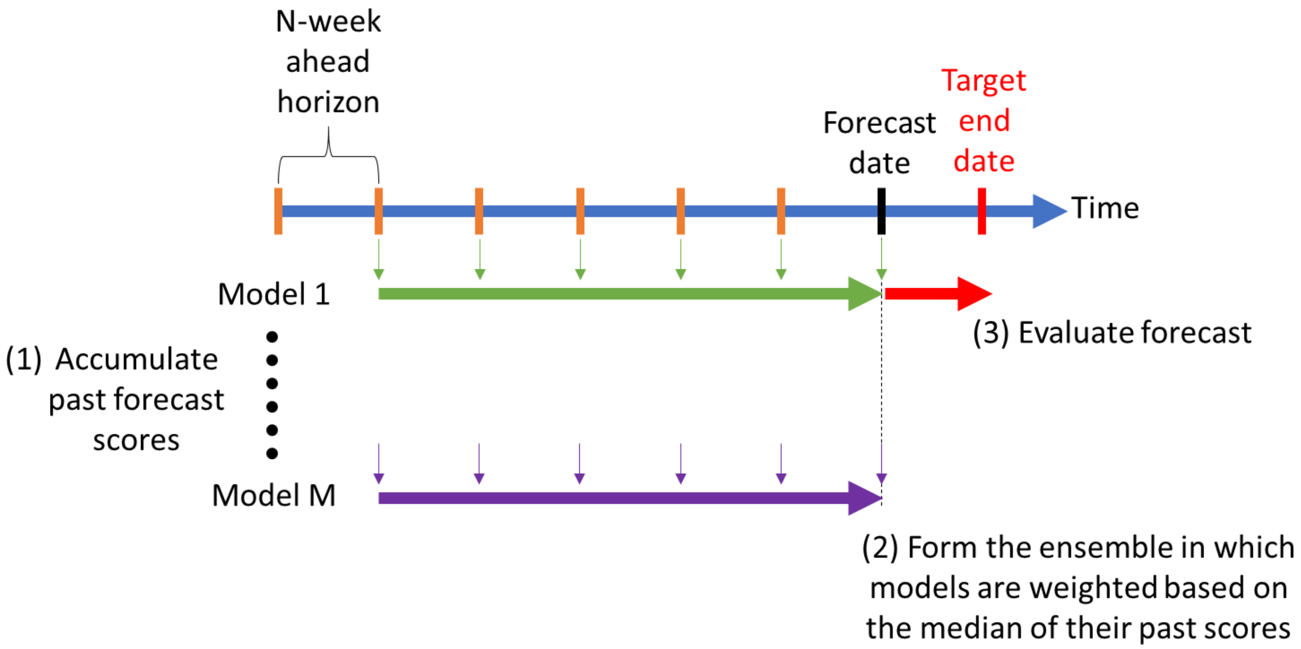

Fig. S4. Ensemble forecast formation.

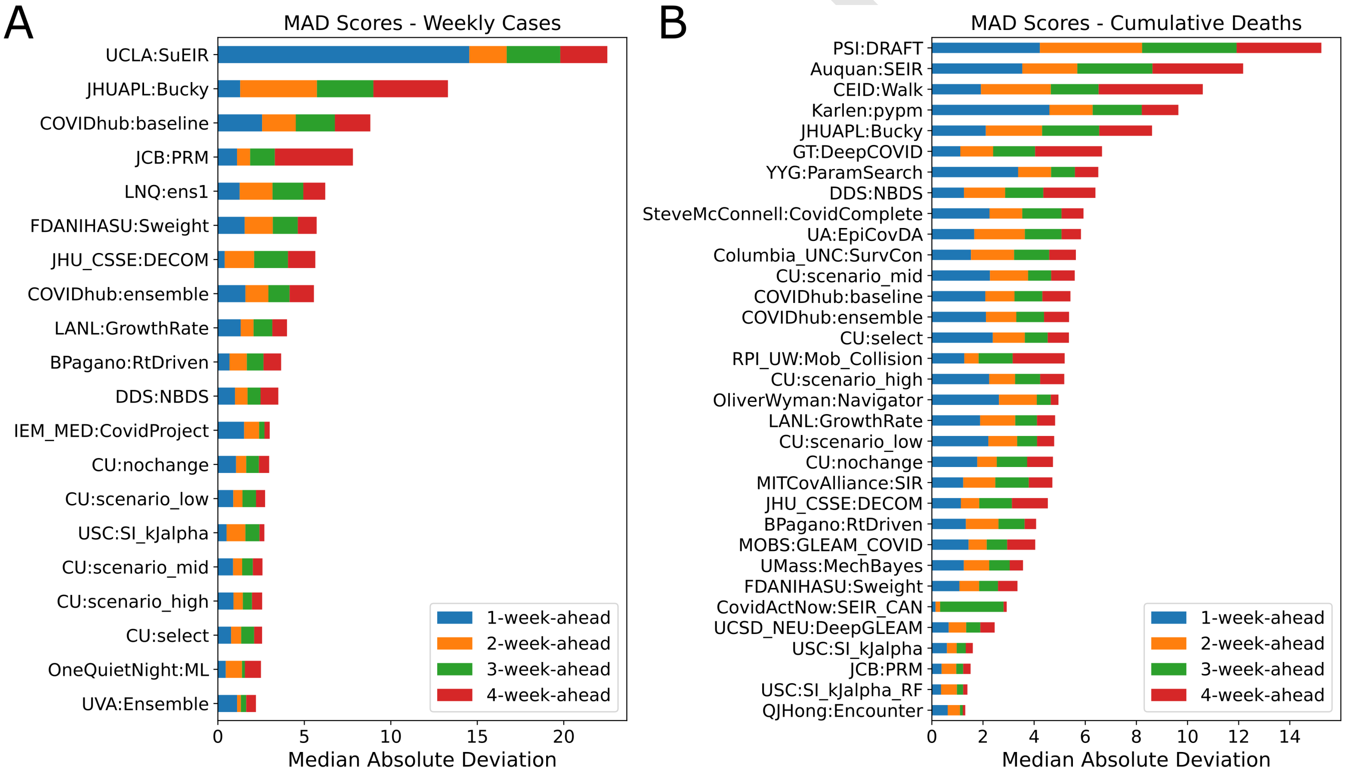

Fig. S5. Median absolute deviation (MAD) as a score variability measure for the models. A. MAD for weekly incidental case forecasts over 1-4-week-ahead forecasting horizon. B. MAD for cumulative death count forecasts over 1-4-week-ahead forecasting horizon. FDANIHASU model is the score-weighted ensemble presented in this work. Note: Models that have at least one ∞ as their MAD in 1- to 4-week-ahead forecasts are not shown in these plots.

medRxiv preprint doi: https://doi.org/10.1101/2021.02.06.21251276; this version posted February 8, 2021. The copyright holder for this preprint (which was not certified by peer review) is the author/funder, who has granted medRxiv a license to display the preprint in perpetuity.
This article is a US Government work. It is not subject to copyright under 17 USC 105 and is also made available for use under a CC0 license.

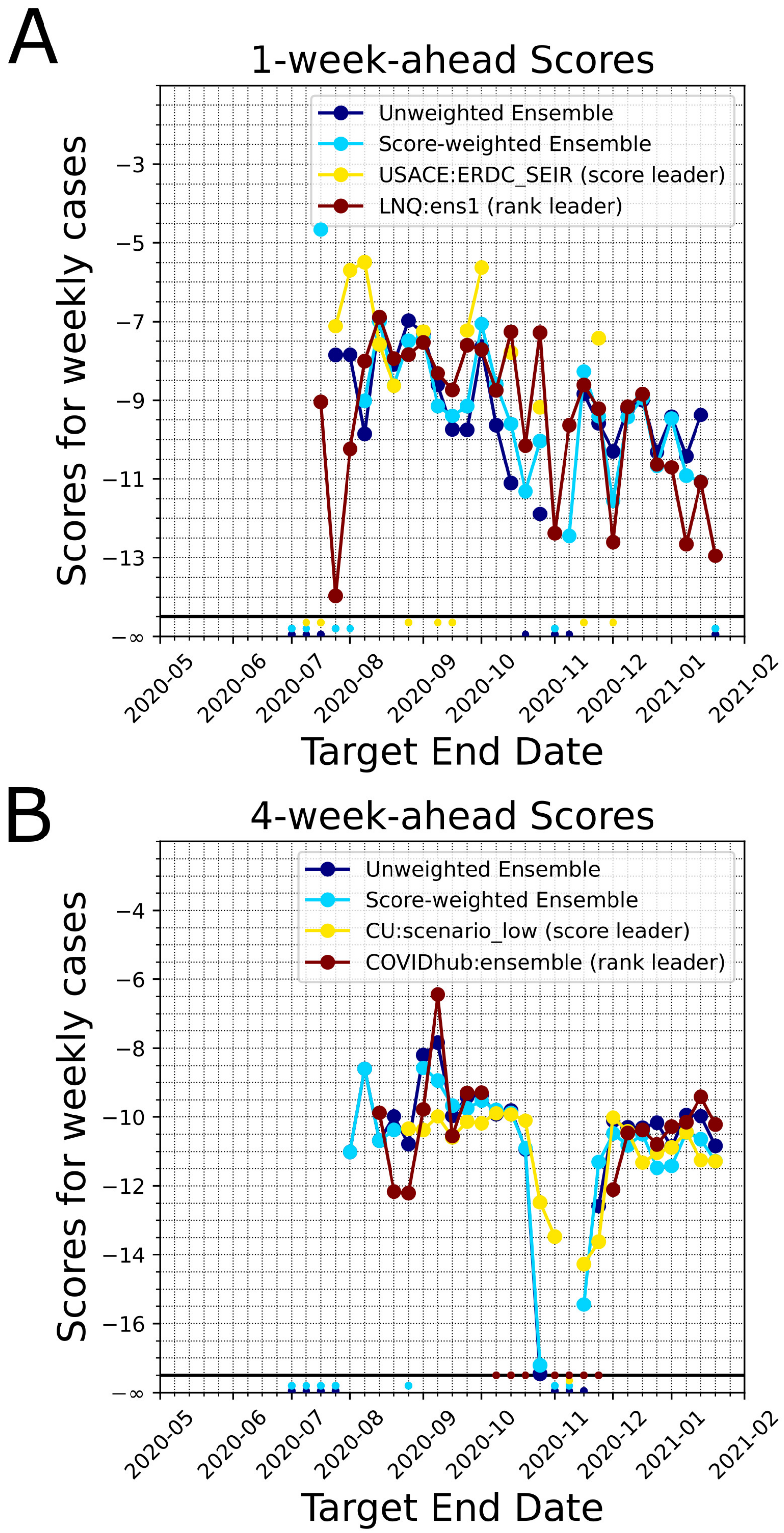

Fig. S6. Comparison of scores of unweighted and score-weighted ensemble models for the weekly incidental case counts. For comparison purposes we plot the leading model's score (based on past performance as of the last target end date, by ranking and by median scores). Model forecasts that do not encompass the ground truth G would have a score $-\infty$ and this scenario is shown at the bottom of the figure panels. A. 1-week-ahead scores. B. 4-week-ahead scores.

medRxiv preprint doi: https://doi.org/10.1101/2021.02.06.21251276; this version posted February 8, 2021. The copyright holder for this preprint (which was not certified by peer review) is the author/funder, who has granted medRxiv a license to display the preprint in perpetuity. This article is a US Government work. It is not subject to copyright under 17 USC 105 and is also made available for use under a CC0 license.

Score Matrix

SCORE	July 4	July 11	...	January 9	January 16	Median
Model 1	-∞	-7.3	...	-2.6	-4.7	-4.3
Model 2	NA	-∞	...	-4.7	-6.5	-5.6
...	...	...	...	...	...	...
Model N-1	NA	NA	...	-∞	-7.8	-17.6
Model N	-5.1	-4.3	...	-∞	-8.9	-18.7

Rank Matrix

RANK	July 4	July 11	...	January 9	January 16	Mean
Model A	7	5	...	1	3	4.3
Model B	NA	10	...	4	4	4.5
...	...	...	...	...	...	...
Model M	NA	NA	...	11	13	17.3
Model N	3	2	...	12	13	18

Fig. S7. Example score and rank matrices for n-Week forecast horizon. Cells in the matrices with NA represent the absence of a forecast for that date. There are 12 categories and each category has its own leader board: 2 for targets (weekly cases, cum deaths) and 6 for time horizons (1-week-ahead,.., 6-week-ahead). We have two conditions in place when forming the leader boards using these matrices. First, the leader boards consider the time frame July 4, 2020 onward. Second, in the leader boards, we do not include models with number of forecasts less than 50% of the number of possible weeks in the time frame.
